# Integrative and Conjugative Elements (ICEs) in Pasteurellaceae Species and Their Detection by Multiplex PCR

**DOI:** 10.3389/fmicb.2018.01329

**Published:** 2018-06-26

**Authors:** Michal Beker, Simon Rose, Claus A. Lykkebo, Stephen Douthwaite

**Affiliations:** Department of Biochemistry and Molecular Biology, University of Southern Denmark, Odense, Denmark

**Keywords:** antibiotic resistance, veterinary macrolides, *Pasteurella*, *Mannheimia*, genomics

## Abstract

Strains of the Pasteurellaceae bacteria *Pasteurella multocida* and *Mannheimia haemolytica* are major etiological agents of bovine respiratory disease (BRD). Treatment of BRD with antimicrobials is becoming more challenging due to the increasing occurrence of resistance in infecting strains. In Pasteurellaceae strains exhibiting resistance to multiple antimicrobials including aminoglycosides, beta-lactams, macrolides and sulfonamides, the resistance determinants are often chromosomally encoded within integrative and conjugative elements (ICEs). To gain a more comprehensive picture of ICE structures, we sequenced the genomes of six strains of *P. multocida* and four strains of *M. haemolytica*; all strains were independent isolates and eight of them were multiple-resistant. ICE sequences varied in size from 49 to 79 kb, and were comprised of an array of conserved genes within a core region and varieties of resistance genes within accessory regions. These latter regions mainly account for the variation in the overall ICE sizes. From the sequence data, we developed a multiplex PCR assay targeting four conserved core genes required for integration and maintenance of ICE structures. Application of this assay on 75 isolates of *P. multocida* and *M. haemolytica* reveals how the presence and structures of ICEs are related to their antibiotic resistance phenotypes. The assay is also applicable to other members of the Pasteurellaceae family including *Histophilus somni* and indicates how clustering and dissemination of the resistance genes came about.

## Introduction

Bovine respiratory disease (BRD) is a common and complex form of pneumonia that affects beef cattle around the world. The disease can be caused by infection with several different viral and bacterial pathogens and is often exacerbated by environmental factors including stress (Taylor et al., 2010). In addition to the distress suffered by infected animals, BRD causes considerable economic losses to the beef cattle industry due to reduced meat yields and outlay associated with preventive measurements and treatment (Griffin, 1997). Numerous studies have therefore focused on identifying the major pathogens implicated in BRD in efforts to reduce animal morbidity and concomitant financial losses. Two of the most common bacterial species associated with this disease have been shown by microbiological and serological surveys to be *Mannheimia haemolytica* and *Pasteurella multocida* (Lillie, 1974; Welsh et al., 2004; Portis et al., 2012), both of which are members of the Pasteurellaceae family.

As with many other bacterial infections, antimicrobial regimens used to combat BRD have been compromised by resistance to numerous drugs including macrolides, aminoglycosides, beta-lactams, and sulfonamides (Kehrenberg et al., 2001, 2005; San Millan et al., 2009; Desmolaize et al., 2011b; Olsen et al., 2015). Macrolide resistance is of particular interest in *P. multocida* and *M. haemolytica* due to the varied mechanisms by which resistance can be conferred via point mutations in ribosomal operons (Poehlsgaard et al., 2012; Olsen et al., 2015) and by acquisition of a range of macrolide resistance genes (Desmolaize et al., 2011a,b; Kadlec et al., 2011). Collectively, resistance genes tend to be clustered within integrative and conjugative elements (ICEs) in the chromosomes of Pasteurellaceae. Macrolide resistance genes include *erm*(42) and *msr*(E)*-mph*(E) and can be acquired in various combinations within an ICE where they are generally interspersed amongst genes conferring resistance to other classes of drugs (Desmolaize et al., 2011a,b; Michael et al., 2012b). Each ICE encodes the machinery required to regulate is own excision, integration, and conjugative transfer (Burrus and Waldor, 2004; Carraro and Burrus, 2015), potentially to other Pasteurellaceae pathogens including *Haemophilus somni* (DeDonder et al., 2016), *Haemophilus parasuis* (Lei et al., 2017), and *Haemophilus influenzae* (Mohd-Zain et al., 2004; Dimopoulou et al., 2007; Juhas et al., 2007, 2013). Previous studies emphasize that Pasteurellaceae species can possess a broad repertoire of antimicrobial resistance genes together with the means of disseminating them (Desmolaize et al., 2011a,b; Michael et al., 2012b), and that the use of appropriate therapeutic agents is therefore of great importance in treating livestock illnesses including BRD.

Detection and characterization of ICE sequences would serve as an initial step in ascertaining whether Pasteurellaceae strains are likely to be equipped with multiple resistance determinants. Due to their large size and diverse composition, the identification of ICEs has up to now required whole genome sequencing coupled with bioinformatics analysis. Sequence characterization of ICE*Pmu1* in *P. multocida* (Michael et al., 2012a,b) and ICE*Mh1* in *M. haemolytica* (Eidam et al., 2013, 2015) revealed structures of 82 and 92 kb, respectively containing core- and accessory gene regions. The core regions encode genes for ICE maintenance and proliferation ensuring the essential functions of transfer, replication, regulation and integration. The accessory regions encode a range of additional traits including antibiotic resistance, metal-fixation and novel metabolic capacities (Wozniak and Waldor, 2010; Bi et al., 2012). The findings from the two *P. multocida* and *M. haemolytica* isolates gave the first clear indication that certain core region features could be common amongst Pasteurellaceae ICEs, whereas the antimicrobial resistance genes and their distribution within ICEs could differ significantly.

Here we report a more comprehensive picture of Pasteurellaceae ICE structures. Initially, we sequenced the genomes of 10 independent *P. multocida* and *M. haemolytica* isolates eight of which were selected due to their resistance to macrolides and in most cases to an array of other veterinary antimicrobials. For strains that had acquired exogenous resistance genes, these were located in nearly all cases within chromosomally-encoded ICEs. Alignment and comparative analysis of the chromosomes revealed sets of core genes common to ICEs and with highly conserved sequences. Conserved genes from different locations within the core regions were chosen to design PCR primer pairs for a multiplex assay to detect the presence of ICEs. Additional primer sets that target rRNA genes located outside the ICE sequences were included in the assay as a means of identifying false negatives and to differentiate between *P. multocida* and other gammaproteobacterial species. Here we describe the application of this assay on over 70 *P. multocida* and *M. haemolytica* isolates, many of which are resistant to multiple drugs. The composition of ICE antimicrobial resistance genes is shown to be highly variable, and while most regions connected with maintenance and transfer are conserved, a significant proportion of the *M. haemolytica* ICEs have lost genes essential for intercellular transfer. *In silico* interrogation of genomes presently available in databases using the multiplex primer sequences shows that the assay is applicable to other Pasteurellaceae members including *Histophilus somni* and confirms that ICE sequences are to be found throughout within this family of bacteria.

## Materials and methods

### Bacterial strains, growth, and macrolide resistance phenotypes

The *P. multocida* and *M. haemolytica* strains are field isolates obtained from nasal swabs of cattle suffering from BRD in Europe and USA, and were procured from the MSD Animal Health culture collection. Strains were plated onto agar containing brain-heart infusion broth (Oxoid, England) and grown at 37°C overnight; cell colonies were purified by restreaking on agar and were then grown again to form individual colonies for direct PCR testing. Standard CLSI procedures (Clinical and Laboratory Standards Institute, 2008) were applied to determine the minimal inhibitory concentrations (MICs) of antibiotics. The macrolides used were tilmicosin (TIL, Sigma-Aldrich, Germany), tildipirosin (TIP, MSD Animal Health, Germany), and gamithromycin (GAM) and tulathromycin (TUL), respectively extracted and purified from Zactran® (Merial, Germany), and Draxxin® (Pfizer, USA). Tildipirosin, gamithromycin and tulathromycin were purified as colorless powders; their structures were verified by liquid chromatography/mass spectrometry and nuclear magnetic resonance.

### Genome sequencing

Eight *P. multocida* and *M. haemolytica* isolates Pmu3358, Pmu3361, Pmu12591, Pmu12601, Pmu14424, Mh6055, Mh12540, and Mh12565 that exhibited intermediate and high resistance to macrolides, plus two macrolide susceptible strains Pmu4407 and Mh11935, were selected for genome sequencing. Genomic DNA was prepared as previously described (Desmolaize et al., 2011a) and sequenced by a paired-end, shotgun approach using Illumina HiSeq equipment (BaseClear, Leiden, Netherlands). Sequence reads were assembled using the CLC genomics workbench (www.CLCbio.com) and the SSPACE premium scaffolder (Boetzer et al., 2011). Assembled contigs were aligned to the reference genomes *P. multocida* 36950 (Genbank: CP003022.1) and *M. haemolytica* 42548 (Genbank: CP005383.1) using the Multiple Genome alignment software, Mauve (Darling et al., 2010) and the genomes were annotated using RAST—Rapid annotation using subsystem technology (Overbeek et al., 2014). Integrative and conjugative elements were identified and their structural similarities were investigated using the bioinformatics tools Island viewer (Langille and Brinkman, 2009), Artemis sequence visualization (Rutherford et al., 2000), Artemis Comparison Tool ACT(Carver et al., 2005) and CMG—Comparative Microbial Genomic software (Vesth et al., 2013).

### Multiplex PCR analyses

Four oligodeoxynucleotide primer pairs were designed to detect genes specific for ICE core regions (Table 1). These primers comprised: pInt1F/pInt1R that bind to *int1* gene encoding a 35.6 kDa integrase and result in a PCR product of 301 bp; pInt2F/pInt2R that give a product of 215 bp and detect a second 29.9 kDa integrase encoded by *int2*; pRelF/pRelR that bind to an ICE-specific relaxase gene (*ICE-rel1*, but not to the homologous *ICE-rel2*) to give a 437 bp product; and pParBF/pParBR that detect *parB* with a product of 503 bp.

**Table 1:** Oligonucleotide primers used for detection of Pasteurellaceae ICE sequences.

**Primer**	**Sequence (5′-3′)**	**Direction**	**Screening function**	**PCR fragment size (bp)**
p84	GACGGAAAGACCCCGTGAACCT	Forward	*rrl* sequence G2053 to T2074 in Gammaproteobacteria	
p85	GGCAAGTTTCGTGCTTAGAT	Reverse	*rrl* sequence A2753 to C2772 in Gammaproteobacteria	720
p86	GGAGCAGCCCCAATCAATCA	Reverse	*rrl* sequence T2633 to C2652 specific to *P. multocida*	600
pParBF	GCTTGGCTCTTCATTGCTCG	Forward	*parB*	
pParBR	TTTCTCCTCCTTGTTGGCGA	Reverse	*parB*	503
pRelF	GGCTCACGTTGGTTTGCTTG	Forward	*ICE-rel1*	
pRelR	TCAGCGGCAGTTTTGCTAAC	Reverse	*ICE-rel1*	437
pInt1F	TAGAACGGAATCATAGACCTGCC	Forward	*int1* (35.6 kDa integrase)	
pInt1R	TGGATTTGCCTTTCTGTTAGTAGT	Reverse	*int1*	301
pInt2F	TCAACATTTCCACATCGTGCTC	Forward	*int2* (29.9 kDa integrase)	
pInt2R	AAGAGGACAGCCAATGAGCC	Reverse	*int2*	215

*The ParB-primers screen for the parB gene; the Rel-primers amplify a region within an ICE-specific relaxase gene, ICE-rel1; and the pairs of Int-primers target two distinct integrase genes int1 and int2. The p85 and p86 primers in combination with the p84 primer are included to verify whether the PCR reaction has functioned (they give a product that is independent of the presence of ICE sequences) and additionally serve to differentiate between P. multocida from other species in the Gammaproteobacteria class. The PCR reactions produce easily distinguishable gel bands ranging from 215 to 720 bp*.

In addition to the ICE-specific primers, three 23S rRNA gene-specific primers, p84, p85 and p86, were included in the multiplex assay (Table 1), and served as internal controls to check whether each PCR assay had functioned and to distinguish between *P. multocida* and other gammaproteobacterial species (Rose et al., 2012). Primers p84 and p85 are complementary to sequences that are conserved in the 23S rRNAs of all Gammaproteobacteria and give a PCR product of 720 bp for bacterial species including *P. multocida, M. haemolytica*, and enterics such as *E. coli*, with no product being formed for species outside the Gammaproteobacteria class. The p86 oligo is specific for *P. multocida* 23S rRNA genes and acts as a nested primer in combination with p84 to produce a 600 bp PCR fragment (Rose et al., 2012). Each of the 75 strains in the study was additionally tested with an independent multiplex assay to detect macrolide resistance genes *erm*(42), *msr*(E), and *mph*(E) (Rose et al., 2012).

Cell colonies were transferred from agar plates and resuspended in 100 μl water, boiled for 5 min, and 1 μl was taken for PCR analyses. Each ICE-specific multiplex PCR was carried out with 200 μM dATP, dCTP, dGTP, and dTTP, 1.0 U *Taq* polymerase (VWR International), 0.4 μM of the primers in 25 μl total volume of 10 mM Tris-HCl pH 8.3, 50 mM KCl, 3.5 mM MgCl_2_, 0.1% Triton X-100. A Mastercycler Personal apparatus (Eppendorf) was used with a denaturation step for 2 min at 95°C followed by 30 cycles of 30 s at 95°C, 30 s at 62°C, 1 min at 72°C, with the final cycle concluding after 5 min at 72°C. PCR fragments were analyzed on 2% agarose gels and the sizes were estimated from a GeneRuler 100 bp DNA Ladder (Fermentas). The multiplex PCR assays for *erm*(42), *msr*(E), and *mph*(E) (Rose et al., 2012) were carried out under the same conditions with primer hybridization at 60°C.

## Results

### Genome analyses

**Figure 1:**
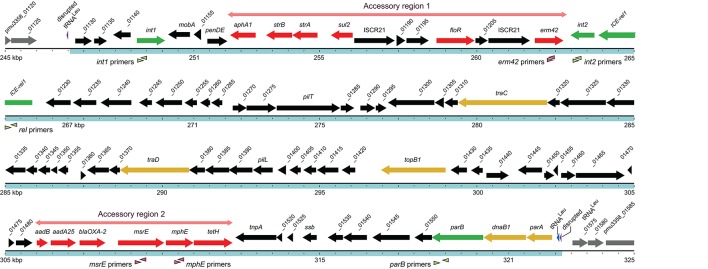
Structure of the 75.6 kb ICE within the chromosome of *P. multocida* strain 3358 is depicted by the blue region, with the extent of the two accessory regions indicated in pale red. The size marker dots are spaced at 500 bp. The *int1, int2, ICE-rel1*, and *parB* genes in the core regions are shown in green, other essential core genes in yellow, the remaining core genes in black, and antibiotic resistance genes in the accessory regions in red. Genes are annotated according to their known functions or by using the NCBI notation (Supplementary Table S1). All numbered genes are prefixed with pmu3358_ as shown for the first and last genes in this figure. The hybridization sites for the primers used in the multiplex PCR assays are indicated by the arrow heads below the respective genes.

**Figure 2:**
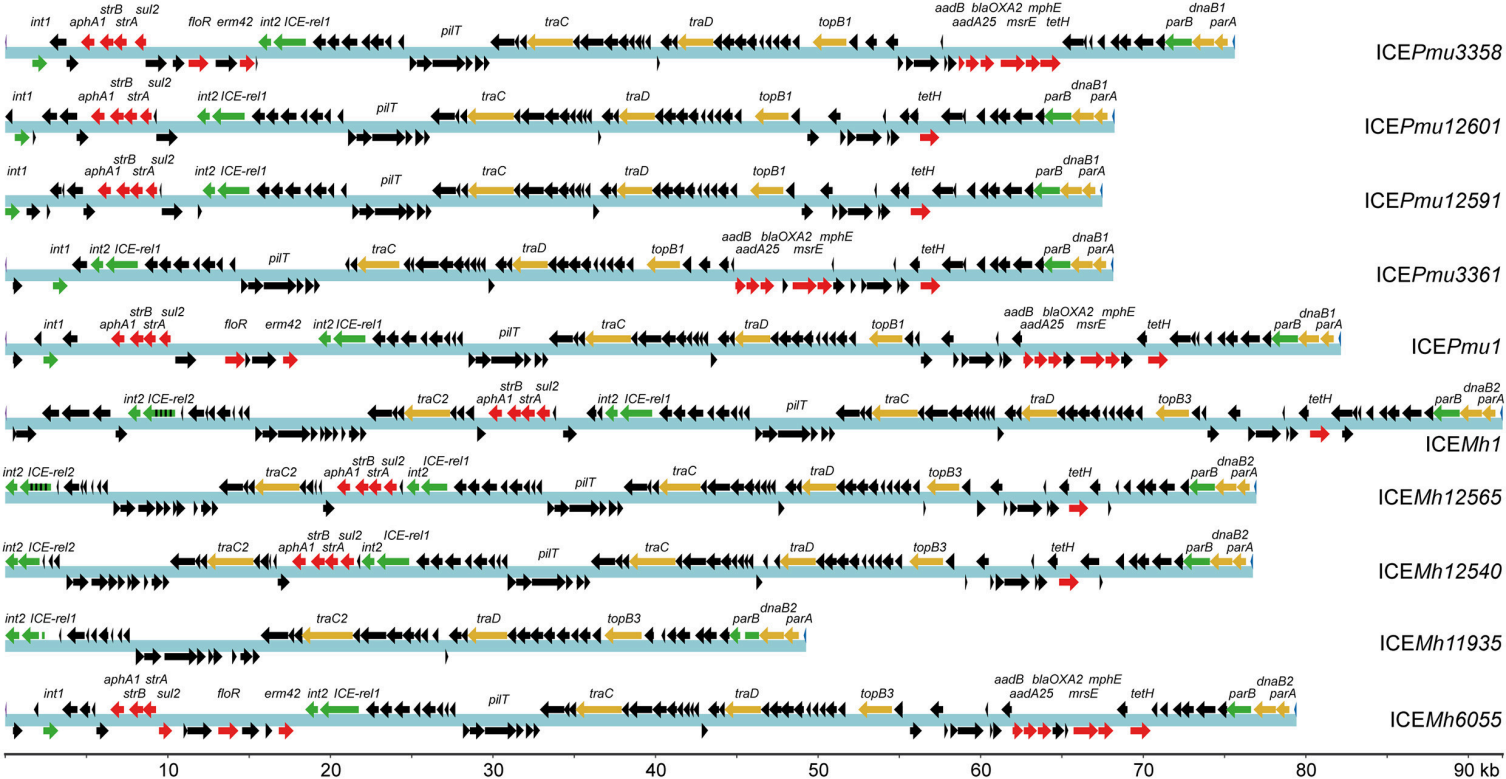
Content of ICEs from the Pasteurellaceae strains determined by whole genome sequencing. The previously reported ICE*Pmu1* of *P. multocida* strain 36950 (Michael et al., 2012b) and ICE*Mh1* from *M. haemolytica* strain 42548 (Eidam et al., 2015) are included for comparison. The ICEs are annotated here according to the strains containing them, and their sizes can be read from the kilobase pair (kb) scale at the bottom. Color coding of the genes as in Figure 1 with the core genes targeted in the multiplex assay (green), other essential core genes (yellow), antibiotic resistance genes (red) plus additional open reading frames (black). The chromosomal site of ICE insertion left a disrupted tRNA^Leu^ gene at both ends of the sequence, which is replaced by in all cases by an intact copy of the tRNA^Leu^ gene located after *parA* (far right, in this sequence orientation). The multiplex assay was design to give a positive signal for *ICE-rel1* but not for the *ICE-rel2* gene (green/black stripes) that is evident in some *M. haemolytica* ICEs. The sensitive strain Pmu4407 contains no ICE genes. Strain Mh11935 also completely lacks resistance genes but contains numerous ICE core region genes, although key genes for ICE transfer and maintenance are missing. The *ICE-rel1* gene in ICE*Mh11935* is truncated and presumably inactive, despite giving a positive multiplex PCR signal. The *parB* gene of ICE*Mh11935* is also truncated, and lacks one of the priming regions needed for a PCR signal. All of the four targeted genes *int1, int2, ICE-rel1*, and *parB* were 100% conserved in ICE*Pmu3358*, ICE*Pmu3361*, ICE*Pmu12591*, and ICE*Pmu12601* of the *P. multocida* strains. Accession codes for the whole genome sequences are listed in Supplementary Table S1.

The genome sizes of the six *P multocida* strains fell within the range 2.24–2.33 Mb, with the smallest genomes belonging to Pmu4407 and Pmu14424 (2,243,572 and 2,245,345 bp, respectively) and the largest to Pmu3361 (2,321,102 bp). The genomes of the four *M. haemolytica* strains were between 2.56 and 2.69 Mb, where Mh11935 possessed the smallest (2,562,294 bp), and Mh6055 the largest (2,689,542 bp). These sizes are within the span of genome lengths considered typical for the two species; for instance, the *P. multocida* strains collectively possessed a core genome of 1,896 genes and these constituted between 88 and 91% of the total genes. The differences in about half of the remaining *P. multocida* genes reflected the presence or absence of an ICE, and this was also the case for the larger *M. haemolytica* genomes. The sizes of the ICEs ranged from 49 to 79 kb, and the most extensive of these were found in *P. multocida* strain 3358 (Figure 1) and *M. haemolytica* strain 6055. Alignment of the sequences showed that they contain regions of similarity with ICE*Mh1* and ICE*Pmu1* (Figure 2). The ICEs in strains Pmu3358 and Mh6055 showed the highest identity with ICE*Pmu1*. Despite the general similarity between the strains, several gaps were evident in the alignments and corresponded mainly to the absence of certain resistance genes in the accessory regions 1 and 2 (Table 2).

**Table 2:** Antibiotic resistance genes and their respective ICE locations, determined by whole genome sequencing in this study.

**Accessory region 1**							**Accessory region 2**					
**ICE**	***aphA1***	***strB***	***strA***	***sul2***	***floR***	***erm*(42)**	***aadB***	***aadA25***	***bla_*OXA*−2_***	***msr*(E)**	***mph*(E)**	***tetH***
ICE*Pmu3358*	+	+	+	+	+	+	+	+	+	+	+	+
ICE*Pmu3361*	–	–	–	–	–	–	+	+	+	+	+	+
ICE*Pmu12591*	+	+	+	+	–	–	–	–	–	–	–	+
ICE*Pmu12601*	+	+	+	+	–	–	–	–	–	–	–	+
ICE*Mh6055*	+	+	+	+	+	+	+	+	+	+	+	+
ICE*Mh12540*	+	+	+	+	–	–	–	–	–	–	–	+
ICE*Mh12565*	+	+	+	+	–	–	–	–	–	–	–	+

*The distribution of genes in accessory region 1 indicates a clustering of resistance determinants for aminoglycosides, florfenicols, macrolides, and sulfonamides. Accessory region 2 contained predominately genes conferring resistance against aminoglycosides, beta-lactams, macrolides, and tetracyclines. Strain Mh11935 harbored an ICE without any these resistance genes*.

### Genes associated with ICE core functionality

The most conserved genes are concerned with replication, conjugative transfer, and chromosomal integration of an ICE, and thereby ensuring its maintenance and dissemination. The ICEs examined here (Table 2) each carry the *int2* gene, which encodes a 30 kDa integrase, while the isolates Pmu3358, Pmu3361, Pmu12591, Pmu12601, and Mh6055 have an additional integrase gene, *int1*, which encodes a paralogous integrase of 36 kDa. Both enzymes belong to the XerD-family of tyrosine integrases (Argos, 1986; Esposito and Scocca, 1997; Nunes-Düby et al., 1998). The *int1* gene is located at the outer edge of the ICE flanking accessory region 1, while *int2* is in the opposite orientation on the other side of accessory region 1 (as shown for ICE*Pmu3358*, Figure 1). The genome sequences showed that the larger integrase is absent in Mh11935, Mh12540, Mh12565.

Between the two ICE accessory regions are the conserved relaxase *ICE-rel1* and the *traC-, traD-*, and *traG-*like genes that encode a type IV secretion system associated with conjugative transfer. The genes *topB, parA, parB*, and *dnaB* are also highly conserved and linked with DNA replication, and the latter three genes are grouped outside accessory region 2 at the far end of the ICE (Figure 2). The *M. haemolytica* strains Mh12540 and Mh12565 possesses second copies the *traC-* and *traG-*like genes that are 80% identical to the homologous sequences present in all the ICEs. These *traC-* and *traG-*like paralogs were also evident in the *M. haemolytica* ICE*Mh1* sequence (Eidam et al., 2015), but absent in the *P. multocida* strains sequenced here. A paralogous relaxase gene *ICE-rel2* was seen in *M. haemolytica* strains and sometimes duplicated, but was absent from our *P. multocida* strains. The ICE-rel2 enzyme has 90% sequence identity with ICE-rel1.

All the *M. haemolytica* and *P. multocida* ICE sequences were integrated within a chromosomal tRNA^Leu^ gene, and the disrupted gene is replaced by an intact tRNA^Leu^ copy at the end of the ICE (Figures 1, 2).

### Antimicrobial resistance genes

The greatest diversity in the ICE structures was observed in the accessory regions 1 and 2, which contain genes conferring resistance to antimicrobials including aminoglycosides, beta-lactams, macrolides, phenicols, sulfonamides, and tetracyclines. The number of antibiotic resistance genes in the strains tested here range from zero to twelve, with the greatest abundance of these genes in strain Pmu3358 and Mh6055. The identities and locations of the resistance genes within the respective ICEs are listed in Table 2.

In accessory region 1, the combination of *aphA1-strB-strA-sul2* genes were observed in several different ICEs, and these cluster with *floR* and *erm*(42) in Pmu3358 and Mh6055. In Pmu3361, region 1 is completely absent. In accessory region 2, the compositions of resistance genes also varied, with strains Pmu3358, Pmu3361, and Mh6055 possessing the combination of *aadB-aadA-bla*_*OXA*−2_*-msr(*E*)-mph(*E*)-tetH* genes, while the remaining strains have only *tetH*.

### Primer design for the multiplex PCR system

Genome sequence comparisons showed that the highly conserved regions associated with ICE-functionality would provide a basis for rapid ICE detection using multiplex PCR, while the more variable ICE regions could be used to differentiate between strains. Primers were designed to target three conserved sequences in *parB, ICE-rel1*, and *int2*. A fourth primer pair detected the other integrase gene *int1* that was present in a subset of ICEs. The locations of these target genes are spread through the length of the ICEs (Figure 2). In addition, three 23S rRNA gene-specific oligonucleotide primers, p84, p85, and p86, were included in the assay to serve as internal controls for the PCR assay and to distinguish between *P. multocida* and other gammaproteobacterial species (Rose et al., 2012).

### Direct screening of bacterial colonies by multiplex PCR

**Figure 3:**
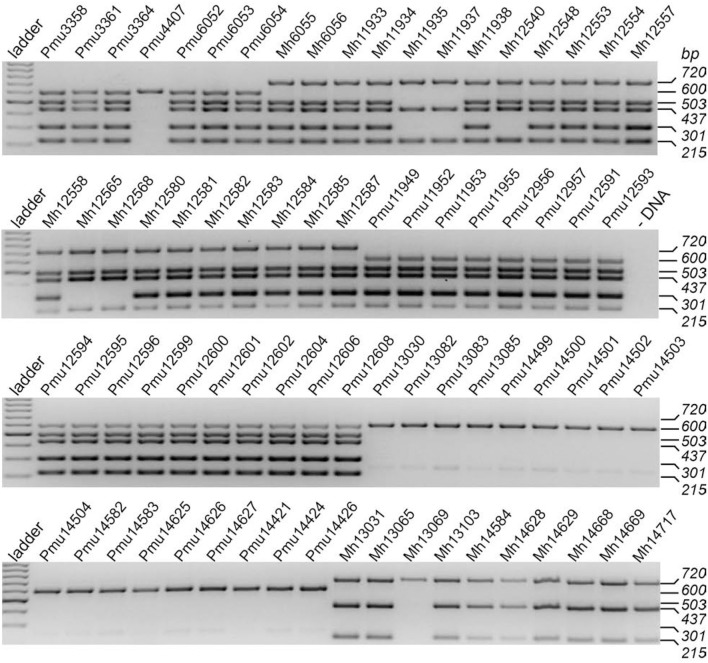
Identification of ICE-related Pasteurellaceae genes using multiplex PCR. The gels show the analyses of 75 field isolates, 43 of which were *P. multocida* (identified by the 600 bp band that is specific for *Pasteurella* spp.) and 32 were *M. haemolytica* (band at 720 bp and lack of 600 bp band). The four ICE-related PCR products correspond to *parB* (503 bp), the ICE-specific relaxase gene (437 bp), and the longer and shorter versions of the integrase genes *int1* (301 bp) and *int2* (215 bp). In strains that lacked all these ICE genes (e.g., Pmu13083) a faint artifact band of 250 bp was sometimes apparent. This band was no longer evident after raising the primer annealing temperature 3°C above the optimal hybridization temperature for the canonical sites. The control lane (-DNA) shows a reaction without template DNA. The bands in the GeneRuler ladder on the left are in 100-bp steps.

A total of 43 *P. multocida* and 32 *M. haemolytica* isolates were screened for ICE sequences using the multiplex assay (Table 3). Twenty-four of the *P. multocida* isolates showed a positive signal for *int1, int2, ICE-rel1*, and *parB*, while the remaining 19 strains produced no signal for any these genes indicating that they lacked an ICE (Figure 3). Several macrolide resistant *P. multocida* isolates were shown to lack all the ICE-specific genes.

**Table 3:** Overview of the *P. multocida* (*Pmu*) and *M. haemolytica* (*Mh*) strains investigated in this study and their macrolide resistance profiles.

**Strains and species**		**Macrolide resistance genes**			**ICE-specific genes**				**MIC**			
		***erm*(42)**	***msr*(E)**	***mph*(E)**	***int1***	***int2***	***ICE-rel1***	***parB***	**TIP**	**TUL**	**TIL**	**GAM**
3358	Pmu*	+	+	+	+	+	+	+	128	>128	128	64
3361	Pmu*	–	+	+	+	+	+	+	2	64	32	32
3364	*Pmu*	–	–	–	+	+	+	+	1	32	16	0.5
4407	Pmu*	–	–	–	–	–	–	–	1	0.5	4	0.5
6052	*Pmu*	+	–	–	+	+	+	+	>128	8	>128	8
6053	*Pmu*	+	–	–	+	+	+	+	128	8	128	16
6054	*Pmu*	+	–	–	+	+	+	+	>128	8	>128	8
6055	Mh*	+	+	+	+	+	+	+	128	>128	128	128
6056	*Mh*	+	+	+	+	+	+	+	>128	>128	>128	128
11933	*Mh*	+	–	–	+	+	+	+	128	8	64	4
11934	*Mh*	+	+	+	+	+	+	+	128	64	128	64
11935	Mh*	–	–	–	–	+	+	–	0.5	2	4	0.25
11937	*Mh*	–	–	–	–	+	+	–	0.5	2	8	0.5
11938	*Mh*	+	+	+	+	+	+	+	128	128	64	128
11949	*Pmu*	+	+	+	+	+	+	+	>128	>128	>128	64
11952	*Pmu*	+	–	–	+	+	+	+	>128	8	>128	8
11953	*Pmu*	+	–	–	+	+	+	+	>128	4	128	4
11955	*Pmu*	+	–	–	+	+	+	+	>128	4	128	4
11956	*Pmu*	+	–	–	+	+	+	+	>128	4	128	4
11957	*Pmu*	+	+	+	+	+	+	+	>128	>128	128	128
12540	Mh*	–	–	–	–	+	+	+	1	2	32	0.5
12548	*Mh*	–	+	+	+	+	+	+	0.5	128	32	64
12553	*Mh*	–	+	+	+	+	+	+	1	128	32	128
12554	*Mh*	–	+	+	+	+	+	+	1	128	32	128
12557	*Mh*	–	+	+	+	+	+	+	1	128	32	128
12558	*Mh*	–	+	+	+	+	+	+	2	>128	32	128
12565	Mh*	–	–	–	–	+	+	+	2	2	32	1
12568	*Mh*	–	–	–	–	+	+	+	2	2	32	1
12580	*Mh*	+	–	–	+	+	+	+	>128	16	128	8
12581	*Mh*	+	–	–	+	+	+	+	>128	16	128	8
12582	*Mh*	+	–	–	+	+	+	+	>128	16	128	8
12583	*Mh*	+	–	–	+	+	+	+	>128	16	128	8
12584	*Mh*	+	+	+	+	+	+	+	>128	128	128	128
12585	*Mh*	+	–	–	+	+	+	+	>128	16	128	8
12587	*Mh*	+	+	+	+	+	+	+	>128	>128	>128	128
12591	Pmu*	–	–	–	+	+	+	+	1	0.5	32	0.5
12593	*Pmu*	–	+	+	+	+	+	+	2	>128	32	64
12594	*Pmu*	–	+	+	+	+	+	+	2	>128	32	64
12595	*Pmu*	–	+	+	+	+	+	+	4	>128	32	64
12596	*Pmu*	–	+	+	+	+	+	+	4	>128	32	64
12599	*Pmu*	–	–	–	+	+	+	+	1	1	32	0.5
12600	*Pmu*	–	–	–	+	+	+	+	1	1	32	0.5
12601	Pmu*	–	–	–	+	+	+	+	1	0.5	32	0.5
12602	*Pmu*	–	+	+	+	+	+	+	4	>128	32	32
12604	*Pmu*	–	–	–	+	+	+	+	1	0.5	32	0.5
12606	*Pmu*	+	+	+	+	+	+	+	>128	>128	>128	128
12608	*Pmu*	+	–	–	+	+	+	+	>128	8	>128	16
13030	*Pmu*	–	–	–	–	–	–	–	0.5	0.5	1	0.25
13031	*Mh*	–	–	–	–	+	+	–	0.25	1	2	*nd*
13065	*Mh*	–	–	–	–	+	+	–	0.25	1	2	*nd*
13069	*Mh*	–	–	–	–	–	–	–	0.5	1	4	*nd*
13082	*Pmu*	–	–	–	–	–	–	–	0.5	*nd*	*nd*	*nd*
13083	*Pmu*	–	–	–	–	–	–	–	0.25	*nd*	*nd*	*nd*
13085	*Pmu*	–	–	–	–	–	–	–	0.5	*nd*	*nd*	*nd*
13103	*Mh*	–	–	–	–	+	+	–	0.25	0.5	1	0.25
14499	*Pmu*	–	–	–	–	–	–	–	4	4	16	2
14500	*Pmu*	–	–	–	–	–	–	–	4	4	16	2
14501	*Pmu*	–	–	–	–	–	–	–	8	4	32	2
14502	*Pmu*	–	–	–	–	–	–	–	8	4	32	2
14503	*Pmu*	–	–	–	–	–	–	–	8	4	32	2
14504	*Pmu*	–	–	–	–	–	–	–	8	4	32	2
14582	*Pmu*	–	–	–	–	–	–	–	8	2	16	1
14583	*Pmu*	–	–	–	–	–	–	–	16	4	16	2
14625	*Pmu*	–	–	–	–	–	–	–	8	0.5	16	0.5
14626	*Pmu*	–	–	–	–	–	–	–	32	16	32	4
14627	*Pmu*	–	–	–	–	–	–	–	32	16	32	4
14421	*Pmu*	–	–	–	–	–	–	–	>64	>64	>64	>64
14424	Pmu*	–	–	–	–	–	–	–	>64	>64	>64	>64
14426	*Pmu*	–	–	–	–	–	–	–	>64	>64	>64	>64
14584	*Mh*	–	–	–	–	+	+	–	16	4	32	4
14628	*Mh*	–	–	–	–	+	+	–	>64	32	64	16
14629	*Mh*	–	–	–	–	+	+	–	>64	32	64	32
14668	*Mh*	–	–	–	–	+	+	–	16	64	8	32
14669	*Mh*	–	–	–	–	+	+	–	8	32	8	8
14717	*Mh*	–	–	–	–	+	+	–	>64	>64	>64	>64

*Strains up to Pmu12608 were isolated in the USA from cattle suffering from respiratory infections (with the exception of Pmu4407, France); strains Pmu13030 to Mh14717 are from cases of bovine respiratory infection in Europe. The genomes of the strains marked with an asterisk were sequenced. All strains were screened using the multiplex PCR assay described here for ICE genes, and additionally with an independent multiplex assay to detect macrolide resistance genes (Rose et al., 2012). The presence (+) or absence (–) of the ICE-specific int1, int2, ICE-rel1, parB genes and the macrolide resistance genes erm(42), msr(E), and mph(E) is indicated. Some of the macrolide resistant strains, such as Pmu14421, Pmu14424, and Pmu14426 that lack ICE structures, have attained resistance via A2059G mutations in all six of their rrn operons; strain Mh14717 has the A2058G mutation in all six of its rrn operons (Olsen et al., 2015). The minimal inhibitory concentrations (MICs in μg/ml) of the macrolides tildipirosin (TIP), tulathromycin (TUL), tilmicosin (TIL), and gamithromycin (GAM), some of which have been reported previously (Rose et al., 2012; Olsen et al., 2015), are color coded to indicate whether the strains show susceptibility (green), intermediate resistance (orange), or resistance (red) according to CSLI breakpoints for these antibiotics: http://vet01s.edaptivedocs.info/GetDoc.aspx?doc=CLSI%20VET01S%20ED3:2015&scope=user. nd, not determined*.

In the *M. haemolytica* isolates, 17 gave positive signals for *int1, int2, ICE-rel1*, and *parB*, and a further three isolates contained *int2, ICE-rel1* and *parB* (but not *int1*). Eleven of the strains gave clear signals for *int2* and *ICE-rel1*, but *parB* was missing (Figure 3), suggesting that these strains possess remnants of ICEs which lack the ability to be disseminated. The multiplex assay was designed to give a positive signal for *ICE-rel1*, but not for the homologous *ICE-rel2* gene present in *M. haemolytica* strains.

The occurrence of the macrolide resistance genes *erm*(42), *msr*(E), and *mph*(E) (or subsets thereof) in the *M. haemolytica* isolates correlates with the presence of ICE sequences containing both *int1* and *int2*. Several *M. haemolytica* isolates with ICE remnants were also macrolide resistant without having any of the *erm*(42), *msr*(E), or *mph*(E) genes (Table 3). Only one *M. haemolytica* isolate tested here (Mh13069) possessed none of the four ICE-specific genes.

## Discussion

The *P. multocida* and *M. haemolytica* strains included in this study were isolated in the USA and Europe from cattle with respiratory tract infections. Strains were initially selected on the basis of macrolide antibiotic resistance. However, the majority of strains associated with BRD did not exceed breakpoint values for macrolides, and some susceptible strains from the same locations were included in this study. Genome sequence analyses of a subset of the strains revealed a series of highly conserved genes that could serve as markers for the presence of an ICE and from these the *int1, int2, ICE-rel1*, and *parB* genes, which are distributed along the length of the ICE structures (Figure 1), were chosen for the multiplex PCR assay.

All the *P. multocida* and *M. haemolytica* strains that possessed one or more of the macrolide resistance genes *erm*(42)*, msr*(E), and *mph*(E) also had a full complement of the *int1, int2, ICE-rel1*, and *parB* marker genes. The converse was not true, however, and five *P. multocida* strains possessed all four ICE marker genes but none of the macrolide resistance genes (Table 3). As previously reported, the different combinations of the *erm*(42)*, msr*(E), and *mph*(E) genes conferred distinct resistance phenotypes to the 15-membered ring macrolides GAM and TUL and the 16-membered macrolides TIL and TIP (Rose et al., 2012), and a full complement of all three genes was required to attain high level resistance to all these drugs (Table 3).

Some strains possessed neither ICE nor resistance genes but were nevertheless highly resistant to macrolides. In these cases, resistance was achieved via point mutations in the drug binding site on the ribosome, as we have seen previously in the *P. multocida* strains Pmu14421, Pmu14424, and Pmu14426 with A2059G substitutions in the 23S rRNA genes in all six of their *rrn* operons, and the *M. haemolytica* strain Mh14717 which had A2058G mutations in all six of its *rrn* operons (Olsen et al., 2015). This same mechanism could explain the macrolide resistance phenotypes of Canadian *M. haemolytica* isolates that lack all the *erm*(42)*, msr*(E), and *mph*(E) genes (Alexander et al., 2013). In the related Pasteurellaceae species *Haemophilus parasuis*, the A2059G mutation has been reported in an Australia isolate from swine respiratory infection (Dayao et al., 2016).

Several *P. multocida* and *M. haemolytica* strains (including Pmu12599, Pmu12600, Pmu12601, Pmu12604, Mh12540, Mh12558, Mh12565, and Mh12658, Table 3) were sensitive to both GAM and TIP but showed intermediate resistance to TIL, and in one case (Pmu3364) also to TUL. None of these strains possessed any of the *erm*(42)*, msr*(E), and *mph*(E) genes, and they contained variable numbers (either none, two or all four) of the ICE gene markers (Table 3), indicating that their macrolide resistance phenotypes were unrelated to the presence of an ICE. The genomes of two of these strains, Pmu12591 and Pmu12601, were sequenced without revealing any changes in their rRNAs or ribosomal proteins, or other relevant genes that could account for elevated MICs to TIL (Vester and Douthwaite, 2001; Peric et al., 2003). Similar observations for *M. haemolytica* strains with no obvious molecular explanation have been made elsewhere for TUL resistance (Alexander et al., 2013) and also for GAM resistance (DeDonder et al., 2016). Up-regulation of one or more endogenous efflux system could possibly account for these anomalous macrolide phenotypes.

Similar to previous accounts (Michael et al., 2012b; Eidam et al., 2015; Clawson et al., 2016), the ICEs analyzed here contained a broad range of genes encoding resistance to other antibiotics that have been widely used in veterinary medicine. Genes conferring resistance to aminoglycoside, beta-lactam, florfenicol, sulfonamide, and tetracycline were distributed throughout accessory regions 1 and 2, with each specific gene maintaining its particular location within an accessory region. For instance, the *aphA1-strA-strB-sul2* combination, previously identified in plasmids (Hirsh et al., 1989; Yamamoto et al., 1990) are found in the same order in accessory region 1, and in some cases followed by *floR* and *erm*(42) (Table 2). This latter region shares 96% sequence identity with plasmid pPDP9106b (Michael et al., 2012b), suggesting that *floR* and *erm*(42) also descended from plasmids. More common in the ICEs were resistance genes for the older aminoglycoside and sulfonamide drugs, consistent with sulfonamide resistance being one of the traits most often detected in *Pasteurella* and *Mannheimia* isolates (Kehrenberg et al., 2001). Strain Pmu14424 contains no ICE, but nevertheless encodes Sul2 (100% identical to the structure in Figure 1), StrA (94% identical), and TetR (65% identical) within a Tn10 transposon adjacent to an inactive mu-bacteriophage at a different chromosomal location (1842728–1846225) than the site of ICE integration (Figure 1).

In accessory region 2, the *aadB-aadA-bla*_*OXA*−2_ combination appears to have originated from the transposon Tn5706 and is generally flanked by the *tetR* and *tetH* genes (Michael et al., 2012b). Although the full-length *tetH* gene was evident at the same location in all the sequenced ICE structures (Table 2), we only observed remnants of *tetR*. The neighboring *msr*(E)*-mph*(E) pair was previously detected in plasmids (González-Zorn et al., 2005; Gołebiewski et al., 2007) and more recently in the Pasteurellaceae (Desmolaize et al., 2011b; Kadlec et al., 2011). In some strains, the *msr*(E)*-mph*(E) genes are flanked by IS26 elements, while in other strains the IS26 sequences are truncated (Pmu3361) or lost (Pmu3358), which could indicate that these macrolide resistance genes were inserted into accessory region 2 prior to ICE acquisition by these strains. The ICE locations of *erm*(42) and/or *msr*(E)*-mph*(E) (Figure 1) and their incidence in the different isolates (Table 3) indicates that they have been integrated into ICE structures from separate plasmids by independent recombination events.

Insertion of ICEs appears to be guided into the same chromosomal site by one or more phage-like integrases. All the ICE strains in this study possessed the *int2* gene encoding a 30 kDa integrase, and in some strains an *int1* gene encoding a larger 36 kDa enzyme was also present (Table 3). Alignment of these paralogs shows they have 49% amino acid identity, with particularly high conservation of residues within the active site that is common to members of the tyrosine recombinase XerD family (Esposito and Scocca, 1997; Nunes-Düby et al., 1998). These enzymes typically target tRNA genes for site-specific integration events (Reiter et al., 1989) and occur here at a tRNA^Leu^ gene, as initially observed for ICE integration in *H. influenzae* (Dimopoulou et al., 2002). A remnant tRNA^Leu^ anticodon loop sequence of 13 nucleotides (5′-GATTTTGAATCAA) remains at all the *attL* sites after ICE integration (Figure 1), while the *attR* site varies in size between 10 and 13 nucleotides with the sequence 5′-GATTTTGAAT(CAA). An intact tRNA^Leu^ copy is located at the end of the ICE immediately after *parA* (Figure 1), and replaces the disrupted gene.

The isolates studied here show that Pasteurellaceae ICE sequences can vary greatly in size and structure with some strains, exemplified by Pmu3358, possess what appears to be a fully functional ICE that encodes multiple resistance determinants. Despite the overall variations in the sizes and content of the ICEs, the core and resistance genes that remained generally showed >99% sequence conservation in the strains analyzed here. A similar picture emerges from the Pasteurellaceae genomes available at Genbank. Interrogating these Pasteurellaceae genomes *in silico* with the probes used in this study showed that a surprisingly high proportion possesses at least two of the *int1, int2, ICE-rel1*, and *parB* genes (Supplementary Table S2). Furthermore, the key proteins Int2 and ICE-Rel1 are generally 100% identical to those in our strains, and are evident in numerous Genbank sequences from *H. somni* isolates in addition to *P. multocida* and *M. haemolytica* (Supplementary Figure S1).

**Figure 4:**
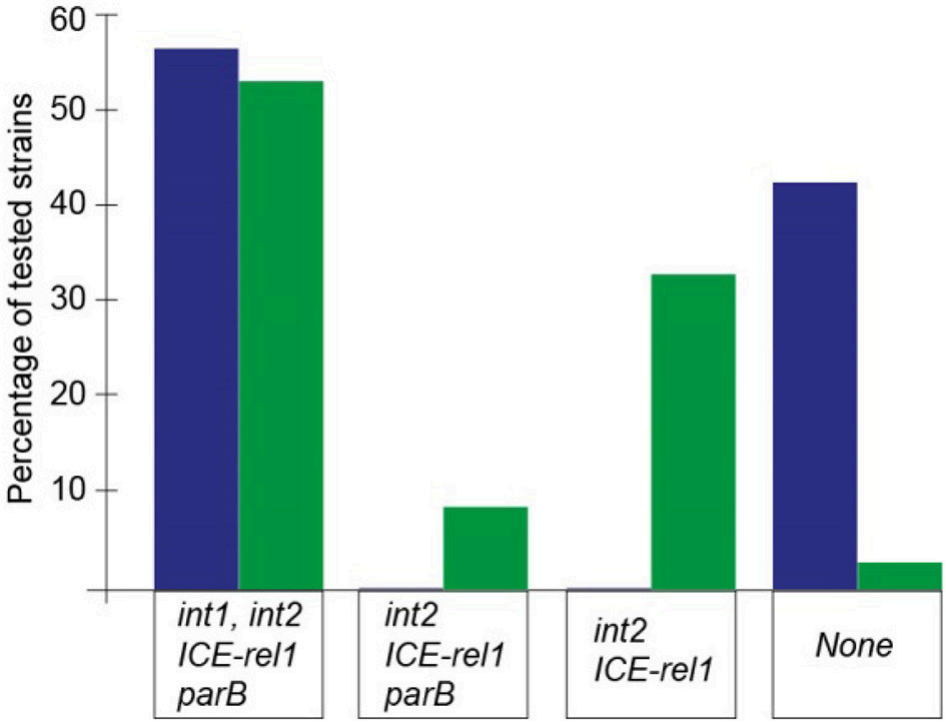
Relative numbers of the *P. multocida* (blue) and *M. haemolytica* strains (green) containing the ICE core genes screened in the multiplex PCR assay. Twenty-four of the 43 *P. multocida* isolates gave a positive signal with each of the *int1, int2, ICE-rel1*, and *parB* primer combinations; and a similar proportion of *M. haemolytica* strains (17 of 32) was also shown to contain these four ICE genes. The remainder of the *P. multocida* strains tested lacked all of the *int1, int2, ICE-rel1*, and *parB* genes, but no *P. multocida* strain was found to harbor a truncated, nonfunctional ICE structure. In contrast, 14 *M. haemolytica* strains possessed degenerate ICE sequences that would presumably not be capable of promoting intercellular transfer.

The data presented here on the conservation of key ICE genes suggest that propagation of these sequences is a relatively recent event within the Pasteurellaceae. The differences in ICE sizes found here reflect the plasticity and relatively rapid sequence losses subsequent to ICE acquisition. The compositions of several *M. haemolytica* ICEs (Figure 4), and also those in the database (Supplementary Figure 1), appear to be partially degenerate and incapable of being disseminated. This contrasts with the *P. multocida* ICE sequences (Table 3 and Figure 4) that have retained their function. *In silico* analysis of other *P. multocida* genomes in the database (Supplementary Table S2) present a similar but not identical picture where, although their ICE sequences appear functional, a proportion has lost *int2* while retaining *int1* (Supplementary Figure 1). This could reflect the wider range of hosts, which include swine and poultry, from which these latter strains were isolated. The overall picture suggests that some of the *M. haemolytica* ICE sequences are of older origin and were acquired before those found in *P. multocida*. In the case of the strains isolated from cattle, this is consistent with *M. haemolytica* being a primary etiologic agent associated with BRD (Klima et al., 2016; Snyder et al., 2017).

Several new questions can now be posed about the transfer of resistance genes in Pasteurellaceae. These include why the presence of the macrolide resistance genes *erm*(42), *msr*(E), and *mph*(E) correlates with that of *int1* despite their relatively distant ICE locations. Possibly the acquisition of these macrolide resistance genes is a recent event and the presence of *int1* is indicative of ICE functionality, which has not yet been lost in the sequences that have been transferred more recently. Finally, although lack of detection is not a proof of absence, we note that the *erm*(42), *msr*(E), and *mph*(E) genes have yet to be reported in European (Table 3) (Rose et al., 2012), Australian (Dayao et al., 2016), and Canadian animals (Alexander et al., 2013).

## Author contributions

MB, SR, and CL: methodology and investigation. SD and MB: writing, review and editing. SD: resources, funding acquisition and supervision.

### Conflict of interest statement

The authors declare that the research was conducted in the absence of any commercial or financial relationships that could be construed as a potential conflict of interest.
