# Supplementary material for: Integrative and Conjugative Elements (ICEs) in Pasteurellaceae Species and Their Detection by Multiplex PCR
Source: Front Microbiol. 2018 Jun 26;9:1329. doi: 10.3389/fmicb.2018.01329 (PMC6028734; doi:10.3389/fmicb.2018.01329)
Supplement: Supplementary file 1 [file Data_Sheet_1.pdf]

Supplementary Material

Integrative and Conjugative Elements (ICEs) in Pasteurellaceae species and their detection by multiplex PCR

Michal Beker, Simon Rose, Claus Asger Lykkebo, Stephen Douthwaite*

Department of Biochemistry and Molecular Biology, University of Southern Denmark, Campusvej 55, DK-5230 Odense M, Denmark.

Running title: Multiplex PCR detection of Pasteurellaceae ICEs

Key words: Antibiotic resistance; veterinary macrolides; *Pasteurella*; *Mannheimia*; genomics.

*Correspondence to: Stephen Douthwaite: srd@bmb.sdu.dk; Tel +45 6550 2395

TABLE S1

Genomic sequence data for the strains described in this article have been deposited at the NCBI database as BioProject ID: PRJNA433612

Isolate	BioSample	Accession no.
Mh6055	SAMN08512412	CP029638
Mh11935	SAMN08512413	CP026858
Mh12540	SAMN08512414	CP026857
Mh12565	SAMN08512415	CP026856
Pmu3358	SAMN08512318	CP029712
Pmu3361	SAMN08512319	CP026861
Pmu12591	SAMN08512320	CP026860
Pmu12601	SAMN08512321	CP026859
Pmu14424	SAMN09094600	CP029322

TABLE S2 *In silico* screening for Pasteurellaceae ICEs in Genbank.

Bacterial species	Strain	Int1	Int2	ICE-Rel1	ParB
P. multocida	36950	+	+	+	+
P. multocida	USDA-ARS-USMARC-60494	+	+	+	+
P. multocida	USDA-ARS-USMARC-60717	+	+	+	+
P. multocida	USDA-ARS-USMARC-60213	+	+	+	+
P. multocida	USDA-ARS-USMARC-60380	+	+	+	+
P. multocida	USDA-ARS-USMARC-60713	+	+	+	+
P. multocida	USDA-ARS-USMARC-60224	+	+	+	+
P. multocida	USDA-ARS-USMARC-60712	+	+	+	+
P. multocida	USDA-ARS-USMARC-60675	+	+	+	+
P. multocida	USDA-ARS-USMARC-59962	+	+	+	+
P. multocida	USDA-ARS-USMARC-60714	+	+	+	+
P. multocida	2125PM	+	-	+	+
P. multocida	2154PM	+	-	+	-
P. multocida	2165PM	+	-	+	+
P. multocida	2267PM	+	-	+	+
P. multocida	2297PM	+	-	+	+
P. multocida	2320PM	+	-	+	-
P. multocida	2335PM	+	-	+	+
P. multocida	2389PM	+	-	+	+
P. multocida	2403PM	+	+	+	+
P. multocida	2428PM	+	+	+	+
P. multocida	2450PM	+	-	+	+
P. multocida	2497PM	+	-	+	+
P. multocida	2512PM	+	-	+	+
P. multocida	2526PM	+	+	+	+
P. multocida	2578PM	+	-	+	+
P. multocida	2597PM	+	-	+	+
P. multocida	2612PM	+	-	+	+
P. multocida	2633PM	+	-	+	+
P. multocida	2668PM	+	-	+	+
P. multocida	2887PM	+	+	+	+
P. multocida	2901PM	+	-	+	+
P. multocida	2930PM	+	-	+	+
P. multocida	2969PM	+	+	+	+
P. multocida	3022PM	+	+	+	+
P. multocida	3045PM	+	-	+	+
P. multocida	3275PM	+	-	+	+
P. multocida	3347PM	+	+	+	+
P. multocida	3384PM	+	-	+	+

P. multocida	3483PM	+	+	+	+
P. multocida	69APM	+	-	+	+
P. multocida	8522APM	+	+	+	+
H. somni	2336	+	-	-	+
H. somni	USDA-ARS-USMARC-63255	+	+	+	+
H. somni	USDA-ARS-USMARC-63368	+	+	+	+
H. somni	USDA-ARS-USMARC-63369	+	+	+	+
H. somni	USDA-ARS-USMARC-63370	+	+	+	+
H. somni	USDA-ARS-USMARC-63374	+	+	+	+
H. somni	3171HS	+	+	+	-
H. somni	3216HS	+	+	+	-
H. somni	3275HS	+	+	+	-
H. somni	3473HS	+	-	-	+
H. somni	3556HS	+	+	+	+
H. somni	3570HS	+	+	+	-
H. somni	3605HS	+	+	+	+
H. somni	3669HS	+	+	+	-
H. somni	3692HS	+	+	+	-
H. somni	3758HS	+	+	+	-
H. somni	3768HS	+	+	+	+
H. somni	3782HS	+	+	+	-
H. somni	3849HS	+	+	+	-
H. somni	3874HS	+	+	+	+
M. haemolytica	M42548	-	+	+	+
M. haemolytica	MhBrain2012	-	+	+	+
M. haemolytica	MhSwine2000	-	-	+	+
M. haemolytica	193	-	-	-	+
M. haemolytica	191	-	-	-	+
M. haemolytica	186	-	-	-	+
M. haemolytica	L02A	-	-	-	+
M. haemolytica	L044A	+	+	+	+
M. haemolytica	L033A	+	-	-	+
M. haemolytica	2125MH	-	-	+	+
M. haemolytica	2165MH	-	+	-	+
M. haemolytica	2190MH	-	+	+	+
M. haemolytica	2297MH	-	+	+	+
M. haemolytica	2428MH	-	+	+	+
M. haemolytica	2436MH	+	+	+	+
M. haemolytica	2512MH	-	+	+	-
M. haemolytica	2543MH	+	+	+	+
M. haemolytica	2578MH	+	+	+	+
M. haemolytica	2597MH	+	+	+	+
M. haemolytica	2612MH	-	+	-	+

M. haemolytica	2633MH	-	-	+	-
M. haemolytica	2683MH	-	+	+	+
M. haemolytica	2700MH	-	-	-	+
M. haemolytica	2769MH	-	+	+	-
M. haemolytica	2887MH	-	-	+	+
M. haemolytica	2930MH	-	-	+	+
M. haemolytica	2969MH	-	+	+	+
M. haemolytica	3022MH	-	+	+	+
M. haemolytica	3045MH	+	+	+	+
M. haemolytica	3369MH	-	+	-	+
M. haemolytica	3384MH	-	-	+	+
M. haemolytica	3483MH	-	-	+	+
M. haemolytica	23209MH	-	+	+	+
M. haemolytica	232020MH	-	-	+	+
M. haemolytica	32754MH	-	+	+	-
M. haemolytica	327511MH	-	-	+	+
M. haemolytica	327518MH	-	+	+	-
M. haemolytica	16041065 BH	-	+	+	+
M. haemolytica	16041065 GH	-	+	+	+
Mannheimia sp.	USDA-ARS-USMARC-1261	+	+	-	+
M. varigena	USDA-ARS-USMARC-1388	+	+	-	-
B. trehalosi	USDA-ARS-USMARC-190	+	+	+	+

TABLE S2 From the genomes that are presently available in databases under the taxonomic ID for Pasteurellaceae species (taxid 712), the 104 strains show here contain one or more of the ICE sequences probed in the PCR multiplex assay. ICE sequences are evident in *Pasteurella multocida* (42 strains with ICE genes out of 109 total strains); *Mannheimia haemolytica* (39/65); *Histophilus somni* (20/22); *Mannheimia varigena* (1/3); *Bibersteinia trehalosi* (1/5); and an unspecified *Mannheimia* isolate (1/1). *H. somni* 2336 is listed in the database as *Haemophilus somnus* 2336. The results of database BLAST searches showing the presence (+) or absence (-) of the ICE-specific proteins Int1, Int2, ICE-Rel1 and ParB are indicated for each of the strains.

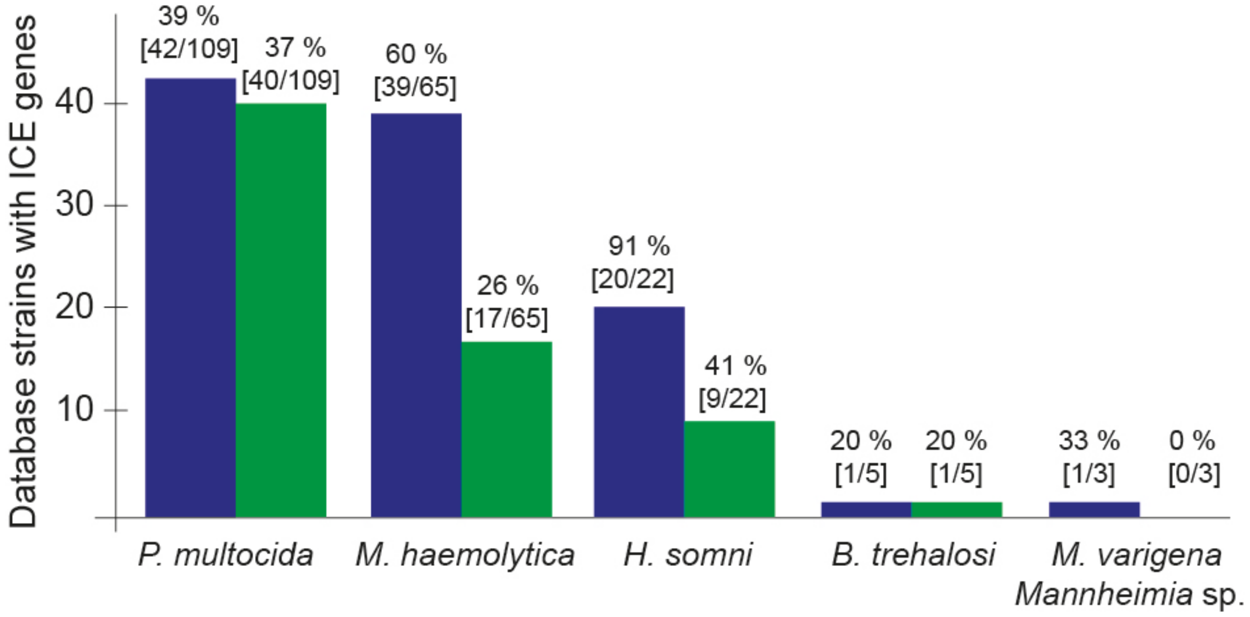

FIGURE S1 Pasteurellaceae genomes that are presently available in databases showing the percentages containing ICE genes. Proportions of the strains containing one or more of the *int1*, *int2*, *ICE-rel1* or *parB* genes are shown in blue. Genomes that contain at least three of these ICE genes including *int1* and/or *int2*, are shown in green. The data include all available sequences, so several strains were isolated from sources other than cattle and thus include swine and poultry pathogens.
